# Age-dependent ferritin elevations and HFE C282Y mutation as risk factors for symptomatic knee osteoarthritis in males: a longitudinal cohort study

**DOI:** 10.1186/1471-2474-15-8

**Published:** 2014-01-08

**Authors:** Lauren Kennish, Mukundan Attur, Cheongeun Oh, Svetlana Krasnokutsky, Jonathan Samuels, Jeffrey D Greenberg, Xi Huang, Steven B Abramson

**Affiliations:** 1 Division of Rheumatology, Department of Medicine, New York University School of Medicine, NYU Hospital for Joint Diseases, New York, NY, USA; 2 Department of Population Health, Division of Biostatistics, New York University School of Medicine, New York, NY, USA; 3 Division of Rheumatology, NYU Hospital for Joint Diseases, 301 East 17th Street, Suite 1410, New York, NY 10003, USA

**Keywords:** Osteoarthritis, Iron, Radiographic progression, Hemochromatosis, Hypoxia inducible factor

## Abstract

**Background:**

Age, gender and genetic predisposition are major intrinsic risk factors for osteoarthritis (OA). Iron increases are associated with age and gene mutation. In the present study, we examined whether serum ferritin, an indicator of total body iron stores, correlates with clinical features in patients with OA, and whether the hemochromatosis Fe (HFE) gene mutation plays a role.

**Methods:**

In a 2-year longitudinal observational study, 127 patients with knee OA and 20 healthy individuals (controls) were enrolled. All patients underwent standardized weight-bearing fixed-flexion posteroanterior knee radiographs. Peripheral blood samples were analyzed for serum ferritin, and genotyped for HFE using allelic discrimination methods.

**Results:**

Higher levels of serum ferritin were found in patients older than 56 years (*P* =0.0186) and males (*P* =0.0006), with a trend toward higher ferritin in patients with OA. HFE gene mutation carriers were more prevalent among patients with OA than among healthy controls. When stratified further by gender, we found that male patients with OA had higher levels of serum ferritin than male control subjects [odds ratio = 4.18 (limits of 95% confidence interval: 0.86–27.69, *P* = 0.048)]. Analyses of radiographic data indicated that higher ferritin was associated with narrower joint space width at baseline (*P* = 0.032) in male patients. Additionally, among men, risk prediction of radiographic severity [Kellgren-Lawrence (KL) grade >2)] in the higher ferritin group was almost five times that of the lower ferritin group (odds ratio = 4.74, *P* = 0.023).

**Conclusion:**

Our data suggest that increased ferritin levels are associated with symptomatic knee OA in males. This finding needs to be validated in a larger cohort of patients.

## Background

Osteoarthritis (OA) is the most prevalent joint disease and a major cause of disability and health care burden [1,2]. It is characterized by progressive loss of joint articular cartilage and subchondral bone remodeling [3]. Advanced imaging studies such as MRI reveal changes of joint space narrowing and cartilage degeneration [3]. The destructive nature of OA is regulated by mediators that break down collagen, proteoglycans and bone in articular joint tissues [4]. Although age, gender, and genetic predisposition are major intrinsic risk factors for the disease [5], the precise clinical and pathophysiological contributors that cause incidence and progression of knee OA remain unknown. Likewise, there is a lack of knowledge regarding biomarkers to predict which patients will develop OA and are more likely to progress to severe disease.

Iron is a part of all living cells, and iron increase is associated with age, gender, and gene mutations. Using the NYU Women’s Health Study, we have found that serum level of ferritin is a reliable biomarker for epidemiological studies [6], and serum ferritin significantly increases during menopausal transition [7,8]. Ferritin is an iron storage protein with a binding capacity of 4,500 atoms of iron per molecule of ferritin [9]. Iron levels are also significantly increased in men during their adolescent years [10,11]. Moreover, hemochromatosis is a common inherited iron overload disorder due to mutations at C282Y and H63D in the HFE (*h*emochromatosis *Fe*) gene [12]. A prevalence of 0.5% C282Y homozygotes is estimated among Caucasians, translating to over one million homozygotes in the U.S. alone [13,14].

While iron is an essential metal for human life, increasing evidence shows that a link between defective iron metabolism and tissue responses may drive the OA phenotype [15]. Indeed, increased levels of iron are found in the synovial fluid and synovial lining cells in OA relative to normal controls [16,17]. Patients with hemophilic arthropathy and hemochromatosis, diseases in which joint tissues are exposed to increased iron levels from hemoglobin or defective iron transport, respectively, resemble OA-like joint degradation [18]. A positive clinical association was established between subclinical H63D mutation in the HFE gene and primary OA in the ankle joints of patients [19], but apparently not for primary hip or knee OA [20]. More recently, it has been shown that hemochromatosis is associated with frequent, early and severe symptoms of arthritis and is a risk factor for knee replacement surgery [21,22].

Iron produces reactive oxygen and nitrogen species, leading to oxidative stress and contributing to OA development [23,24]. Iron also regulates iron-responsive genes through iron regulatory proteins/iron-responsive element (IRE) system [25]. Among eight genes identified to date, a phylogenetically conserved IRE was discovered in the 5′-untranslated region of the hypoxia-inducible factor-2alpha (HIF-2α) mRNA. HIF-2α is essential for endochondral ossification and embryonic skeletal growth, and HIF-2α expression was shown to be higher in osteoarthritic cartilages versus non-diseased cartilages of mice and humans [26]. It has been shown that HIF-2α directly induces the expression in chondrocytes of genes encoding catabolic factors, such as matrix metalloproteinases (MMP1, MMP3, MMP9, MMP12 and MMP13), aggrecanase-1, nitric oxide synthase-2 and prostaglandin-endoperoxide synthase-2 (COX-2) [27]. In the presence of high iron, the level of HIF-2α is upregulated through IRE [28], suggesting that iron-mediated HIF-2α could be a catabolic transcription factor in the osteoarthritic process.

The goal of the present study was to evaluate whether iron levels, as measured by serum ferritin, correlate with clinical characteristics of OA presence and severity, and whether polymorphisms in the HFE gene contribute to OA development and progression. Our data suggest that increased ferritin levels, associated with age, gender, and HFE subtype C282Y gene mutation, may promote cartilage damage with knee OA.

## Methods

### Patient recruitment

One hundred twenty-seven patients with symptomatic knee OA (SKOA) had completed 24-month follow-up when this study was initiated as part of a larger ongoing NIH-funded prospective study of 180 subjects evaluating biomarkers in OA. Patients were recruited at New York University Hospital for Joint Diseases. To be eligible for the parent study, patients had to be at least 40 years old and respond, on an initial phone questionnaire, “yes” when asked if they had knee pain for most of the last month that was relieved by rest, at least partially. All patients were then evaluated for knee OA by history and physical exam and had to fulfill ACR clinical criteria for the diagnosis of knee OA (knee pain plus at least 3 of the following 6 criteria: age >50 years, stiffness <30 minutes, crepitus, bony tenderness, bony enlargement, no palpable warmth) [29]. Exclusion criteria were: any other form of arthritis (including rheumatoid arthritis, spondyloarthritis, active crystal arthropathy); body mass index (BMI) ≥33; any disorder requiring the use of systemic corticosteroids within 1 week of screening; history of bilateral knee replacements; major co-morbidities including diabetes mellitus, non-cutaneous cancer within 5 years of screening, chronic hepatic or renal disease, chronic infectious disease, congestive heart failure; and hyaluronan and/or corticosteroid injection to the affected knee within 3 months of screening. Twenty individuals without OA were enrolled into the study as controls. These subjects had no clinical signs or symptoms of any type of arthritis and were free of knee pain. We assessed pain, functional status and quality of life with the Western Ontario and McMaster Osteoarthritis Index (WOMAC) [30], 36-item Short Form Health Survey (SF-36) [31], and Multidimensional Health Assessment Questionnaire (MDHAQ) [32]; within the MDHAQ are modules for the patient global assessment of their disease and for visual analog scale (VAS) pain assessment. The Institutional Review Board (IRB) of New York University School of Medicine approved the protocol. Informed consent was obtained from all study participants prior to enrollment.

### Knee radiographs

All patients underwent standardized weight-bearing fixed-flexion posteroanterior (PA) knee radiographs using the SynaFlexer™ X-ray positioning frame (Synarc). Radiographic readings were done separately by two musculoskeletal radiologists blinded to patient demographics, clinical information and MRI readings. Disagreements between the two readers were resolved by consensus. X-rays were scored for Kellgren-Lawrence (KL) grade (range: 0–4) [33], and medial and lateral joint space width (JSW) were measured at the mid-portion of the joint space via electronic calipers [34]. We recruited 20 control subjects who had KL radiographic score <1 and no knee pain. All patients were examined by one of two NYUHJD investigators (SK, JS) every 6 months in this 24-month study. Radiographic assessments at baseline and 24 months included bilateral (signal and non-signal knee) KL determination, quantitative measurement of medial and lateral joint space width (JSW), medial and lateral joint space narrowing (JSN), using the OARSI atlas, by two musculoskeletal radiologists blinded to patient information. Cohen’s kappa coefficients for inter-rater agreement for KL scores of the right and left knees were 0.85 and 0.77, respectively, and >0.85 for most other radiographic outcome measures. Concordance correlation coefficients were >0.90 for JSW measurements.

The Osteoarthritis Research Society International (OARSI) atlas was used to determine osteophytes, medial and lateral joint space narrowing, medial tibial/lateral femoral subchondral sclerosis, and medial tibial attrition [35]. Diseased compartment joint space narrowing (JSN) was defined as the JSN score in the compartment designated as more diseased based upon JSW measurements. Radiographic progression was defined as those who had change in KL or narrowing of medial joint space width in signal knee between baseline and 24-month follow-up.

### Serum ferritin measurements

Patients with OA in the sub-study presented here had blood samples taken at baseline and 18-month visits, and healthy control participants at baseline and 24-month visit. Serum was isolated from blood samples. Serum ferritin was measured by ELISA as previously described by our laboratory using human anti-goat ferritin antibody (Meridian Life Science, Inc, OH, USA), human liver ferritin as standards (Calbiochem, CA, USA), and a micro plate reader (Spectramax Plus, Molecular Devices, CA, USA) [7]. The intra- and inter-assay coefficients of variation for ferritin measurements were 9% or less. Ferritin levels in all serum samples of the study were above the detection limit of 1.5 ng/ml and were included in the statistical analyses. At the time of start of this study, baseline serum samples for both control and OA, and 24-month samples for control participants and 18–month samples for patients with OA, were available for ferritin estimation.

### DNA isolation and genotyping

Blood samples (5 ml) were collected (pyrogen-free heparinized tubes) for DNA extraction. Two single nucleotide polymorphisms (SNPs) in the HFE gene (C282Y and H63D) were genotyped. Genotyping for the HFE C282Y and H63D mutations was accomplished by polymerase chain reaction (PCR) using validated commercial SNP primers and probes (ABI TaqMan SNP Genotyping Assays C_1085595_10 and C_1085600_10, Applied Biosystems, CA, USA) along with detection using allelic discrimination computation (ABI Prism 7900HT Detection Systems, Applied Biosystems, CA, USA). Six genotypes were then reported: wild type (WT) or +/+; C282Y heterozygote or C282Y/+; C282Y homozygote or C282Y/C282Y; H63D heterozygote or H63D/+; H63D homozygote or H63D/H63D; and C282Y/H63D compound heterozygote or C282Y/H63D. Final genotypes were scored blinded to patient clinical data.

### Statistical analyses

We first described the patients with respect to demographic factors as well as markers of severity and radiographic symptom of OA. We explored data graphically and numerically to assess the distributions of measurements. Because serum ferritin level was not normally distributed (skewed distribution), ferritin level was assessed either as a continuous variable after a natural logarithm transformation (Ln) or as a dichotomous variable by grouping together at the third quintile of ferritin levels (quintiles 1 + 2+ 3 compared with quintiles 4 + 5). OA severity was defined by JSW as well as KL grade (0–4), and was dichotomized for statistical analyses. Our data were grouped into the following major OA severity categories: mild/moderate (KL 2) and severe (KL 3, 4) based on the KL scale, and dichotomized based on JSW into minor (≤4 mm) and major (>4 mm).

Univariate statistics were calculated for continuous variables, and a Chi-square test was used for categorical variables. Multivariate linear (logistic) regression models with log-transformed serum ferritin values (tertile groups) as independent variables were also used to assess the relations between elevated serum ferritin and the severity and extent of OA progression (as the dependent variable) while controlling for the effects of age, sex, BMI and baseline score of the disease severity variable as covariates. All covariates associated with serum ferritin in the univariate analyses were included in the final multivariate model. OA progression is defined by JSN over 24 months. For each participant, changes in scores for JSW were calculated by subtracting the score at baseline from that at follow-up, so the negative values indicate narrowing or decrease of values. Coefficients and their standard deviation from the linear regression, odds ratios (ORs), adjusted odds ratios (AORs) for age, gender and BMI, and their 95% confidence intervals (CI) from the logistic regression coefficients and their corresponding *P* value for trend were computed. Potential confounding variables known to be associated with response variable were included in multivariable models (age, gender, BMI).

## Results

### Demographic and clinical characteristics of the study participants

The summaries of demographic and clinical characteristics and the differences in these parameters between the SKOA and control subjects are presented in Table 1. The mean age (± standard deviation [SD]) of patients in the OA group was 65.1 (±10.0) years (range 40 to 85 years); 63% were female; and mean BMI was 26.6 (±3.6). In the control group the mean age was 56.1 (±9.1) years (range 43 to 75 years); 45% were female; and mean BMI was 26.6 (±4.1). Patients with OA were significantly older than healthy control participants (*P* = 0.00034). At baseline, levels of serum ferritin were 32% higher in patients with OA (59.1±114.9) compared to control participants (37.1±23.0), although the difference was not statistically significant (p = 0.334). Change in serum ferritin levels between baseline and follow-up were not significant in either the control or OA groups. Furthermore, positive delta change (increased) in serum ferritin levels was observed in control participants, in contrast to negative delta change (decreased) in ferritin levels in patients with OA. These results led us to stratify and further analyze ferritin by age, gender, and gene mutation. HFE genotyping results indicate that there were 95 wild type (+/+), 18 H63D/+ heterozygotes, 7 C282Y/+ heterozygotes, 2 H63D homozygotes, 2 C282Y/H63D compound heterozygotes, and 3 C282Y homozygotes among the patients with OA. In the control group there were 15 wild type (+/+) and 5 H63D/+; no other variants were detected.

**Table 1:** Comparisons of demographic and clinical characteristics of patients with osteoarthritis (OA) and healthy individuals (control) at baseline, and of ferritin levels at baseline and 18-month follow-up

**Variables: All Subjects (*N*** **= 147)**	**Control (*N*** **= 20)**	**OA (*N*** **= 127)**	***P *** Value*
Age (years), mean ± SD	56.1 ± 9.0	65.1 ± 10.0	0.00034**
Female gender, number (%)	9 (45%)	80 (63.0%)	0.1446***
Caucasian race, number (%)	11 (55%)	86 (67.7%)	0.5099***
Ethnicity:			
Hispanic, number (%)	4 (20%)	14 (11.0%)	0.4405***
Non-Hispanic, number (%)	16 (80%)	113 (89.0%)	
BMI (kg/m^2^), mean ± SD	26.6 ± 4.1	26.6 ± 3.6	0.9547**
Genotypes, number (%):			
+/+ (wild type)	15 (75%)	95 (74.8%)	0.7962***
H63D/+	5 (25%)	18 (14.2%)	
C282Y/+	0	7 (5.5%)	
H63D/H63D	0	2 (1.6%)	
C282Y/H63D	0	2 (1.6%)	
C282Y/C282Y	0	3 (2.4%)	
Ferritin (ng/ml), mean ± SD:			
Baseline	37.1 ± 23.0	59.1 ±114.9	0.3344**
18-month follow-up	47.7 ± 34.5	53.3 ± 55.8	0.7747**
Delta	8.8 ± 24.8	-6.6 ± 88.8	0.3317**
**Variables: Male Subjects (*N*** **= 58)**	**Control (*N*** **= 11)**	**OA (*N*** **= 47)**	***P *** Value*
Age (years), mean ± SD	58.4 ± 10.1	67.1 ± 9.7	0.0205**
Caucasian race, number (%)	6 (54.5%)	34 (72.3%)	0.2901
Ethnicity:			
Hispanic, number (%)	2 (18.2%)	14 (8.5%)	0.3178
BMI (kg/m^2^), mean ± SD	27.7 ± 3.5	26.6 ± 3.1	0.3716

*P, comparison between OA group and healthy controls by **two-sample t-test or ***Chi-square test.

Table 2 compares severity and progression of SKOA in female and male patients with OA. Baseline scores for pain VAS and WOMAC did not differ significantly between females and males, and JSN over 24 months was similar in both groups (Table 2).

**Table 2:** Severity of symptomatic knee osteoarthritis (SKOA) according to clinical and radiographic measures: pain visual analog scale (VAS) and Western Ontario and McMaster Osteoarthritis Index (WOMAC) scores at baseline; change in Kellgren-Lawrence (KL) grade from baseline to 24 months; joint space width (JSW) at baseline and 24 months; and progression of joint space narrowing (JSN) at 24 months

**Variables**	**OA (*N*** **= 120)**	**Female (*N*** **= 76)**	**Male (*N*** **= 44)**
VAS Pain, mean ± SD (0-100 mm)	40.21 ± 24.6	42.3 ± 24.5	37.4 ± 29.6
WOMAC, mean ± SD	170.25 ± 105.2	173.6 ± 104.2	164.5 ± 107.6
KL delta from baseline to 24 months, *N* (%)			
-1	3 (2.5%)	2 (2.6%)	1 (2.3%)
0	88 (73.3%)	56 (73.7%)	32 (72.7%)
1	26 (21.6%)	16 (21.1%)	10 (22.7%)
2	2 (1.7%)	2 (2.6%)	0
3	1 (8.3%)	0	1 (2.3%)
JSW at baseline, mean ± SD (mm)	3.272 ± 1.766	3.603 ± 1.58	2.7 ± 1.93
JSW at 24 months, mean ± SD (mm)	2.989 ± 1.761	3.388 ± 1.513	2. 3 ± 1.956
JSN over 24 months, mean ± SD (mm)	0.283 ± 0.996	0.21 ± 0.88	0.4 ± 1.16

### Association of serum ferritin with age, sex, HFE mutation, and OA phenotype

The association of serum ferritin with disease status and demographic features was then evaluated by univariate analysis in 147 subjects including the healthy controls. Table 3 indicates a trend towards increased serum ferritin levels in patients with SKOA relative to healthy control participants. We observed positive relationships between higher serum ferritin with age (older than 56 years, mean of control group) and with males under both continuous and dichotomous analyses (Table 3). When stratified further by gender, we found that male patients with OA had higher levels of serum ferritin as compared to the male control subjects when dichotomized ferritin was used [OR = 4.18 (95% CI: 0.86–27.69, *P* = 0.048 by Chi-square test), not shown in Table]. No significant differences in serum ferritin levels were found among different races. Among the different HFE variants, C282Y homozygote carriers have significantly higher levels of ferritin as compared to other genotype groups (Table 3; *P* = 0.00006).

**Table 3:** Univariate analyses of ferritin levels at entry after natural log (Ln) transformation according to diseases, age, sex, race, and genotype at entry

**Variables**	**Continuous ferritin levels**		**Dichotomized ferritin levels**	
	**Mean ± SD ng/ml**	***P *** value*	**Odds ratio [95% CI]**	P value**
**OA patients (*N*** **= 127)**	59.1 ± 114.9	0.334	2.21 [0.71–8.25]	0.151
**Healthy controls (*N*** **= 20)**	37.07 ± 22.98			
**Age >56 years (*N*** **= 109)**^**¶**^	62.5 ± 121.7	0.0186	2.31 [0.94– 6.15]	0.050
**Age ≤56 years (*N*** **= 38)**	35.6 ± 22.8			
**Female (*N*** **= 89)**	51.94 ± 133.5	0.0006	2.8 [1.34– 5.9]	0.0034
**Male (*N*** **= 58)**	62.49 ± 43.7			
**White (*N*** **= 97)**	57.7 ± 128.9		0.78 [0.37–1.7]	0.595
**Asian (*N*** **= 13)**	48.52 ± 33.9	0.727		
**African American (*N*** **= 37)**	54.4 ± 44.8	0.411		
**Genotype:**				
**+/+ (*N*** **= 110)**	47.4 ± 38.7	Reference		
**H63D/+ (*N*** **= 23)**	43.0 ± 37.1	0.529***	1.2 [0.44–3.38]	0.646
**C282Y/+ (*N*** **= 7)**	44.2 ± 34.8	0.951***	0.65 [0.06– 4.19]	0.709
**H63D/H63D (*N*** **= 2)**	21.3 ± 11.6	0.401***		NA
**C282Y/H63D (*N*** **= 2)**	75.3 ± 6.6	0.210***		NA
**C282Y/C282Y (*N*** **= 3)**	513.6 ± 646.9	0.00006***		NA

Data are presented as mean ± standard deviation (SD).

P values refer to difference between two groups as derived from:

*t test on log-transformed ferritin.

**Chi-square test.

^¶^Cut-off based on mean age of healthy controls.

***+/+ (wild type) versus mutation.

### Association of serum ferritin with SKOA radiographic severity

To determine whether levels of serum ferritin can predict disease severity, cross-sectional analyses were carried out in patients with SKOA according to radiographic disease severity, based on medial JSW and KL grade (KL0/1/2 vs. KL3/4) at baseline (Table 4). Baseline serum ferritin levels were available for 120 patients with SKOA. Similarly as in previous analyses, ferritin was explored as a continuous variable with a natural logarithm transformation and dichotomous variable (with the third quintile ferritin selected as the cutoff: ≤47.2 or >47.2 ng/ml). We found that male patients and the older group (>74 years old) had significantly higher ferritin levels (*P* = 0.0001, 0.047 in Table 4), which was consistent with findings in the general population [36]. Interestingly, when defining radiographic severity based on medial JSW at baseline as a dichotomized variable by <4 mm (the top tertile), the severity group with smaller medial JSW had higher ferritin levels (*P* = 0.032 by t-test). When samples were dichotomized at the third quintile of ferritin levels, the higher ferritin group had a 3-fold greater likelihood of having severe OA (*P* = 0.006). When further adjusted for age and sex in a multivariate model, the statistical significance was retained (*P* = 0.035). When KL was used to define severity (KL2 versus KL3/4), no statistical association was detected (Table 4). However, when data were stratified by sex, it was observed that the risk prediction of radiographic severity (KL >2) among men in the higher ferritin group was almost five times that of the lower ferritin group of men (OR = 4.74, *P* = 0.023, data not shown). This became more significant when adjusted for age (*P* = 0.012). Among women, however, there were no significant differences regarding the risk of radiographic severity (data not shown).

**Table 4:** Univariate and multivariate analyses in the osteoarthritis (OA) population for relationship between serum ferritin level and demographical, clinical, and radiographic variables at entry

**Variables: All OA (*N*** **= 120)**	**Serum ferritin**	***P *** value*	**Odds ratio**	**Adjusted Odds ratio**
	**(ng/ml), mean ± SD**		**[95% CI; *P *** value**]	**[95% CI; *P *** value***]
Sex:				
Female (*N* = 76)	37.79 ±33.3	0.0001	4.1	NA
Male (*N* = 44)	65.3 ±45.0		[CI: 1.76–9.87; *P =* 0.0004]	
Age (years):				
≤74 (*N* = 105)	45.8 ±39.8	0.047	3.48	NA
>74 (*N* = 15)	61.9 ±40.8		[CI: 0.99–14.01; *P =* 0.045]	
JSW (mm) baseline:				
>4 (*N* = 80)	34.4 ±30.0	0.032	3.2	2.66
≤4 (*N* = 40)	54.6 ±42.9		[CI: 1.29–8.78; *P =* 0.006]	[CI: 1.07–6.64; *P =* 0.035]
KL severity baseline:				
KL2 (*N =* 18)	46.3 ±40.6	0.975	1.505	1.63
KL3/4 (*N =* 72)	48.6 ±39.7		[0.459–5.459; *P* = 0.5943]	[0.54–5.00; *P* = 0.3859]

*t test on log ferritin.

**Chi-square test on dichotomized ferritin levels at third quintile.

***Logistic regression with adjustment for body mass index (BMI), age, sex.

### Association of serum ferritin with radiographic progression

Because higher ferritin is associated with narrower medial JSW, we then analyzed whether serum ferritin is a predictor of JSN at 24 months. For radiographic progression studies we dichotomized SKOA patients based on those who had JSN >0.5 mm over 24 months. Table 5 shows that when samples were divided at the third quintile of ferritin level (≤47.2 ng/ml), the higher ferritin (>47.2 ng/ml) group had a 58% lower risk of having severe radiographic progression than the lower ferritin group in univariate analysis (OR = 0.42, *P* = 0.046). This surprisingly negative association was retained (*P* = 0.017) under the multivariate model adjusted for BMI, age and gender. [We adjusted for age, gender and BMI as all three are risk factors for developing OA.] When stratified by gender, the strength of this association is shown to be driven by the female group. This negative association in females suggests that ferritin synthesized during menopausal transition and years after menopause may be in its apo-ferritin form without iron in it, which possesses an antioxidant capacity. However, this awaits further validation with larger sample sizes.

**Table 5:** Univariate and multivariate analysis for association of osteoarthritis (OA) progression [defined by joint space narrowing (JSN)] with serum ferritin (dichotomized at third quintile)

**OA progression variable**	Odds ratio*	Adjusted Odds ratio**
	**[95% CI; *P *** **value]**	**[95% CI; *P *** **value]**
**All (*N*** **= 120)**		
JSN (mm)		
≤ 0.5 (*N* = 82)	0.42	0.32**
> 0.5 (*N* = 38)	[CI: 0.15–1.023; *P =* 0.046]	[CI: 0.12–0.81; *P =* 0.0170]
**Female (*N*** **= 76)**		
JSN (mm)		
≤ 0.5 (*N* = 53)	0.076	0.07***
> 0.5 (*N* = 23)	[CI: 0.001–0.55; *P =* 0.002	[CI: 0.009–0.63; *P =* 0.016]
**Male (*N*** **= 44)**		
JSN (mm)		
≤ 0.5 (*N* = 29)	0.91	0.79***
> 0.5 (*N* = 15)	[CI: 0.21–4.06; *P =* 1.0]	[CI: 0.21–3.02; *P =* 0.74]

*Univariate logistic regression

**Multivariate logistic regression adjusted for body mass index (BMI), age, sex.

***Multivariate logistic regression adjusted for body mass index (BMI), age.

## Discussion

In the present study, we have shown for the first time that elevated iron level in the form of serum ferritin resulted in a 4-fold increased risk of developing OA in males, and higher ferritin is associated with narrower joint space width in male patients. No statistically significant difference was detected between female patients with OA and their control counterparts. As a whole, despite higher serum ferritin levels in patients with OA than in healthy control participants, the difference is not statistically significant (Table 3).

It is known that levels of serum ferritin increase sharply in males toward the end of the adolescent years (18–30 years old) and reach maximum (150 ng/ml) before 40 years of age [11]. In females this occurs during the menopausal transition years (42–51 years old) and plateaus at 100 ng/ml before 70 years of age [8,37,38]. These results suggest that longer exposure to higher iron is a risk factor for SKOA in males. The finding of no significant difference in serum ferritin levels between women with OA and female control participants may be explained in part by the fact that women have shorter exposure to maximum ferritin levels, and that even these maximum levels are lower than those in men, as well as the fact that all female participants in the present study (both OA and controls) were in the age range during which the female body experiences extreme variations in iron levels.

Because the study was designed to evaluate whether iron levels correlate with clinical characteristics of OA presence and progression, we did not purposefully recruit patients with iron overload conditions as other investigators have done [39]. Nonetheless, we found that HFE gene mutation carriers are more prevalent among our patients with SKOA than healthy control participants (Table 1). Despite the small sample size in the cohort, our results are in agreement with previous studies showing that HFE gene mutations are associated with risk for OA development [19,39-43]. Among the H63D and C282Y HFE mutations and their compound heterozygotes, C282Y homozygosity in patients with SKOA resulted in significant increase in serum ferritin, confirming a previous report that C282Y mutation carriers are highly prevalent in hand OA involving metacarpophalangeal joints 2–5 and bilateral specified large joints [39]. Furthermore, a recent study has reported that patients with genetic hemochromatosis are at increased risk for arthropathies, including the need for joint replacement surgery [44].

Our results also suggest that increased iron may be of importance in the joints since increased ferritin is associated with narrower medial JSW in the diseased joint at baseline in male OA patients (Table 4). In our longitudinal analyses, higher ferritin is associated with slower progression of JSN in female OA patients (Table 5). Our data indicate that higher serum ferritin levels in males are associated with increased severity of disease, measured as JSW, in a cross-sectional study. The fact that elevated ferritin levels at baseline do not also correlate with more rapid progression in this cohort may have several explanations. First, risk factors for disease incidence may not also confer increased risk for disease progression, as has been noted for obesity. Alternatively, as Mazzuca et al. have shown [45], progression of joint space narrowing may be inversely related to baseline joint space width. Finally, our sample size is small and further studies in larger populations are needed to confirm this observation. Another possible explanation for the latter observation is that ferritin synthesized in the body during the menopausal transition and years after menopause is apo-ferritin without iron in it, possessing greater anti-oxidant capacities. Thus, higher ferritin in female patients is associated with slower OA progression during the period we monitored.

It is important to note that the study has a number of limitations. First, the relatively small sample sizes for the OA patients and particularly for the control subjects with younger ages have limited our ability to detect more significant association. Second, we measured levels of ferritin in serum but not in the synovial fluid. It has been shown that iron markers are increased in synovial fluids in patients with OA [16,17] and ferritin concentration is 4-fold higher in synovial fluid than in serum [39]. These results suggest that high serum ferritin likely leads to high local ferritin in the synovial fluid and, thus, measurements of local ferritin in the diseased location may be a better marker in predicting knee OA development. Third, we measured ferritin protein, but not ferritin saturation, in the present study. In view of the capacity of one molecule of ferritin to bind up to 4,500 atoms of iron, ferritin is considered a double-edged sword, with a pro-oxidant property by releasing iron when it is more saturated and an antioxidant property by sequestering iron when it is in apo form [46]. In the present study, ferritin may be more saturated in OA patients than in control subjects. However, this was not measured due to lack of reliable assay for ferritin saturation. Finally, large variations of serum ferritin levels in females as a menopausal factor also need to be considered when designing such studies.

## Conclusions

In sum, our present study strengthens previous findings on the association of increased iron with OA incidence. Importantly, we have shown that higher ferritin levels have gender-specific effects in our patients with symptomatic knee osteoarthritis (SKOA). At baseline, higher ferritin levels were associated with narrower joint space width (JSW) in male SKOA patients. Further investigation in a larger cohort using ferritin as a biomarker of OA severity and progression is warranted. Better understanding of the molecular mechanisms of iron-induced OA pathogenesis may have clinical implication for OA treatments.

## Abbreviations

AOR: Adjusted odds ratio; BMI: Body mass index; CI: Confidence interval; COX-2: Cyclooxygenase-2; HFE: Hemochromatosis Fe; HIF-2α: Hypoxia-inducible factor-2alpha; IRB: Institutional Review Board; IRE: Iron-responsive element; JSN: Joint space narrowing; JSW: Joint space width; KL: Kellgren-Lawrence radiographic grade; Ln: Natural logarithm transformation; MDHAQ: Multidimensional Health Assessment Questionnaire; MMP: Matrix metalloproteinase; OA: Osteoarthritis; OARSI: Osteoarthritis Research Society International; OR: Odds ratio; PA: Posteroanterior; PCR: Polymerase chain reaction; SD: Standard deviation; SF-36: 36-item Short Form Health Survey; SKOA: Symptomatic knee osteoarthritis; SNP: Single nucleotide polymorphism; VAS: Visual analog scale; WOMAC: Western Ontario and McMaster Osteoarthritis Index; WT: Wild type.

## Competing interests

All the authors declare that they have no financial and personal relationships with other people or organizations that could potentially and inappropriately influence (bias) their work and conclusions.

## Authors’ contributions

M.A., J.G., X.H. and S.B.A. were involved in the conception and design of the study; L.K., M.A., S.K. and J.S. in acquisition of data; and L.K., C.O., J.G., X.H. and S.B.A. in analysis of data. L.K., M.A. and X.H. drafted the article, and all authors edited and revised it for important intellectual content. S.B.A. and X.H. take responsibility for the integrity of the work as a whole, from inception to finished article. All authors approved the final version to be published.

## Pre-publication history

The pre-publication history for this paper can be accessed here:

http://www.biomedcentral.com/1471-2474/15/8/prepub
